# Motion State Estimation with Bandwidth Constraints and Mixed Cyber-Attacks for Unmanned Surface Vehicles: A Resilient Set-Membership Filtering Framework

**DOI:** 10.3390/s24216834

**Published:** 2024-10-24

**Authors:** Ziyang Wang, Peng Lou, Yudong Wang, Juan Li, Jiasheng Wang

**Affiliations:** College of Mechanical and Electrical Engineering, Qingdao Agricultural University, Qingdao 266109, China; 20222104019@stu.qau.edu.cn (Z.W.); loupeng@qau.edu.cn (P.L.); 20232111029@stu.qau.edu.cn (Y.W.)

**Keywords:** robust state estimation, resilient set-membership filter, unmanned surface vehicle (USV), mixed cyber-attacks, binary coding schemes (BCS), restricted bandwidth

## Abstract

This paper investigates the motion state estimation problem of the unmanned surface vehicle (USV) steering system in wireless sensor networks based on the binary coding scheme (BCS). In response to the presence of bandwidth constraints and mixed cyber-attacks in USV communication networks, this paper proposes an improved set-membership state estimation algorithm based on BCS. This algorithm partially addresses the problem of degraded performance in USV steering motion state estimation caused by mixed cyber-attacks and bandwidth constraints. Furthermore, this paper proposes a robust resilient filtering framework considering the possible occurrence of unknown but bounded (UBB) noises, model parameter uncertainties, and estimator gain perturbations in practical scenarios. The proposed framework can accurately estimate the sway velocity, yaw velocity, and roll velocity of the USV under the concurrent presence situation of mixed cyber-attacks, communication capacity constraints, UBB noises, model parameter uncertainties, and estimator gain perturbations. This paper first utilizes mathematical induction to provide the sufficient conditions for the existence of the desired estimator, and obtains the estimator gain by solving a set of linear matrix inequalities. Then, a recursive optimization algorithm is utilized to achieve optimal estimation performance. Finally, the effectiveness of the proposed estimation algorithm is verified through a simulation experiment.

## 1. Introduction

With the development of networked system and communication technology, the utilization of unmanned surface vehicles (USVs) is becoming increasingly significant in the supervision of marine ranches. Acquiring the real-time state of USV steering motion is crucial for enabling functions such as vigilant patrolling and intelligent obstacle avoidance. The states (or variables) of USV steering motion mainly include rudder angle, yaw velocity, roll velocity, and sway velocity, which can provide references for USV to make navigation decisions and path planning. However, direct measurement of these data requires the use of expensive sensors, which are highly susceptible to corrosion and failure in sea environments, resulting in the inability of USVs to function [1]. To address this problem, the use of soft measuring techniques to estimate the state of USV steering motion has attracted research interest in both academia and industry [2,3,4].

In fact, state estimation has been a research hotspot in various fields such as fault diagnosis [5,6,7], control engineering [8,9,10], and signal processing [11,12,13] during the last few decades. Among these hotspots, USV is one of the research subjects, attracting a growing interest from researchers. For example, in [14], an augmented state adaptive Kalman estimator was designed to estimate the state and disturbances of an underactuated USV. In the case of a USV experiencing packet loss and delays, a fault-detection filter based on an observer was designed to estimate possible faults and disturbances of the USV [15]. It is important to point out that the current findings regarding state estimation of the USV are mostly obtained based on the assumption that the noise belongs to $L_{2}\left[0,\infty \right)$ or follows Gaussian distribution [16,17,18]. However, in practical operation, USVs are subject to disturbances from wind and waves, and these disturbances have statistical characteristics that are unknown but bounded (UBB). In response to these practical situations, the traditional state estimation approaches, such as Kalman filtering and $H_{\infty }$ filtering, become inapplicable. While set-membership filtering has unique advantages in dealing with UBB noise, its application in USVs is very limited. We have only seen two published research results [19,20], and these existing studies have not yet considered the problems of uncertainty in real sea conditions.

It is well known that uncertainty widely exists in practical systems. For USVs, on one hand, the steering motion model of the USV overlooks certain minor factors during the modeling process, resulting in a certain degree of uncertainty in the model itself. On the other hand, as the USV’s hull is immersed in seawater for extended periods, the components on the USV are inevitably subjected to corrosion from seawater and sea breeze, leading to performance degradation and aging phenomena, which can cause perturbations in model parameters, subsequently introducing perturbations in the estimator gains for state estimation [21]. The occurrence of these two situations can deteriorate the estimation performance of the USV system’s motion state. Therefore, the resilience of state estimation as a practical requirement when designing estimators. In recent years, the problem of resilient/non-fragile set-membership estimation for various systems has gained significant attention from scholars. For instance, In [22], a approach based on non-fragile set-membership estimator was proposed to estimate the quantized discrete-time memristive neural networks with mixed time-delay under the maximum error first protocol, which can effectively reduce the influence of estimator gain perturbations on estimation performance. In [20], a resilient set-membership estimation approach based on the Try-Once-Discard (TOD) protocol was proposed for the discrete model of USV steering motion with uncertain parameters, which ensures that the estimation errors are confined to predetermined ellipsoidal regions even in the presence of uncertain parameters. In practice, although USVs may simultaneously experience estimator gain perturbations and parameter uncertainties, based on our knowledge, we have not seen research results that consider estimator gain perturbations and parameter uncertainty simultaneously, let alone designing corresponding resilient/non-fragile algorithms. In addition to the aforementioned uncertainty problems, the application of wireless communication networks brings communicative conveniences for USV, while also presenting potential threats [23,24].

One potential threat of network communication is that, due to the open and unprotected nature of USV communication networks, unauthorized individuals such as illegal fishermen or competitors can easily obstruct the normal transmission of data by carrying out cyber-attacks, which may cause the USV estimation performance to deteriorate. Therefore, the timely detection and correction of potential cyber-attacks in measurement data of USV have gained increasing attention. For example, in [15], a fault-detection filter based on the elastic event-triggered mechanism was proposed to mitigate the influence of Denial of Service (DoS) attacks on the USV while reducing the communication burden. In [25], an event-based distributed edge-triggered extended state observer was designed to estimate the underactuated USV subjected to DoS attacks, which enables the timely detection of cyber-attacks and the quick recovery of the USV’s position and speed information when the attacks disappear. However, it is worth noting that, on the one hand, considering the types (e.g., bias injection attack [26], replay attack [27], DoS attack [28], and mixed attacks [29]) and features (e.g., intermittency [30], probability [31], and occasionality [32]) of cyber-attacks are various, it is challenging to consistently detect them correctly. On the other hand, considering the complex and variable nature of real sea conditions, even if an attack signal is detected correctly, it may still cause some irreversible influences on the estimation performance if it is not handled in a timely manner. Therefore, unlike the existing “passive” solutions to cyber-attack problems, a seemingly natural idea is to select an “active” solution to mitigate the influence of the cyber-attacks on communication channels, thereby enhancing the state estimation performance of USVs in response to cyber-attacks. This is also one of the original intentions in this study.

Another potential threat of network communication is that, due to bandwidth constraints in channels, network-induced phenomena such as missing measurements and time-delays may occur during data transmission, leading to a decrease in the overall performance of the estimator [33,34,35,36,37,38]. Various approaches have been proposed to reduce the communication burden, thereby avoiding the occurrence of the aforementioned network-induced phenomena [19,20,39,40,41]. For instance, in the problems of USV heading tracking under wave disturbances, a coding scheme based on logarithmic quantization was proposed to reduce communication burden and mitigate data conflicts [19]. In [20], a collective state estimation algorithm based on the TOD scheduling protocol was proposed for USV steering motion in bandwidth-constrained networks, which allows only a sensor node with the maximum difference from the previous values to send the latest data to the networks at each transmission time, thereby reducing communication burden while ensuring that estimation errors are confined within predetermined ellipsoidal regions. From the aforementioned literature and related studies, it can be observed that existing studies either utilize various network protocols and triggering mechanisms to schedule sensors or employ coding scheme based on logarithmic quantization to compress data in the channels. These approaches effectively reduce the communication burden to a certain extent, but in practical engineering, the quality of the transmitted data is difficult to guarantee due to reasons such as excessive number of sensor nodes, improper triggering threshold setting and overly complex logarithmic quantization processes. Additionally, it is also challenging for these existing approaches to effectively mitigate the influence brought by cyber-attacks. Therefore, the main motivation of this study is to select a suitable solution for the steering motion state estimation problem of USV with communication channels influenced by the mixed cyber-attacks and bandwidth constraints, which ensures the quality of the data transmitted, while not only “actively” mitigating the influence of cyber-attacks on communication channels, but also reducing the communication burden.

To address the above-mentioned problem, considering the robustness and simplicity of binary data in transmission [42,43,44,45,46], we propose to utilize binary coding scheme (BCS) to encode the data, thereby mitigating the influence of bandwidth constraints and cyber-attacks on the state estimation performance of USV steering motion. In addition, to cope with the potentially changing of network bandwidth, a binary bit string length selection strategy has been proposed and introduced into BCS. As a popular active security measure, BCS has gained extensive application in networked systems for the purpose of ensuring dependable data transmission [39,47,48,49]. However, when it comes to the USV steering motion, the state estimation problem based on the BCS has not received sufficient attention under the framework of set-membership estimation, this is mainly due to the following challenge: the implementation of the BCS enhances the complexity of the model, which poses significant challenges to the designs and performance analysis of the estimator.

Based on the above discussions and practical requirements, this paper proposes a robust resilient filtering framework for the problem of USV steering motion state estimation based on BCS under the situations of mixed cyber-attacks, channel bandwidth constraints, UBB noises, parameter uncertainties, and estimator gain perturbations. The primary contributions of this paper are: (1) it is proposed that applying the BCS to the communication channel of the USV, which is threatened by cyber-attacks and bandwidth constraints, to reduce the communication burden, actively mitigate the influences of cyber-attacks on data transmission; (2) the considered mixed cyber-attacks consist of the bias injection attack and DoS attack, where the DoS attack is continuous and bias injection attack are stochastic, which better reflects the actual situation of the engineering; (3) the uncertainty problems, such as parameter uncertainties and estimator gain perturbations, are considered simultaneously, and a new framework of resilient set-membership estimation is designed for both cases.

The subsequent sections of this paper are organized in the following way: Section 2 gives the modeling of the USV steering motion model, the specific steps of the BCS and the design of the resilient set-membership state estimator, respectively. Section 3 provides sufficient conditions for the existence of the desired estimator by examining the solvability of a set of recursive Linear Matrix Inequations (LMIs) and obtains the locally optimal estimator gain by a recursive optimization algorithm. Section 4 verifies the algorithm’s effectiveness via a simulation experiment. Section 5 gives the conclusion of the paper.

**Notation.** $R^{h\times n}$ denotes the h×n real constant matrix. $R^{n}$ denotes the n-dimensional Euclidean space. $P\{\beta_{o}=1\}=\beta$ means the probability of the stochastic variable $\beta_{o}=1$ occurring is β. “T”denotes the transpose of a matrix. The notation S≥Z(S>Z) means that S−Z is positive semi-definite (positive definite), where *S* and *Z* are the symmetric matrices. diag{•} indicates a diagonal matrix. Other notations used are quite standard.

## 2. Problem Formulation

### 2.1. Modeling of the USV Steering Motion Model

In practical situations, we can analyze the USV steering motion from six degrees of freedom: yaw, pitch, roll, heave, sway, and surge. If we consider the asymmetrical motion [16], we can only focus on three main degrees of freedom: roll, yaw, and sway. The influence of pitch, heave, and surge can be treated as the disturbance. Then, we can obtain basic equations for sway, yaw, and roll based on Newton’s second law:

(1) $$\left\{\begin{array}{c} i_{xx_{k}}\frac{d^{2}F_{k}}{dt^{2}}=k_{k},\;\;\;\;roll\\ i_{zz_{k}}\frac{d^{2}H_{k}}{dt^{2}}=n_{k},\;\;\;yaw\\ M_{y_{k}}\frac{d^{2}y_{k}}{dt^{2}}=f_{y_{k}},\;\;sway \end{array}\right.$$

where $y_{k}$, $x_{k}$ and $z_{k}$ denote the transverse axis (directed to starboard), normal axis (directed from top to bottom), and longitudinal axis (directed from aft to fore), respectively; $n_{k}$ and $k_{k}$ denote the moments with respect to the $z_{k}$ and $x_{k}$ axes, respectively; $i_{zz_{k}}$ and $i_{xx_{k}}$ denote the moments of inertia with respect to the $x_{k}$ and $z_{k}$ axes, respectively; $M_{y_{k}}$ denotes the effective mass of the marine vehicle in the $y_{k}$ direction; $F_{k}$, $H_{k}$, and $f_{y_{k}}$ denote the roll angle, the heading angle, and the force in the $y_{k}$ direction, respectively.

**Figure 1:**
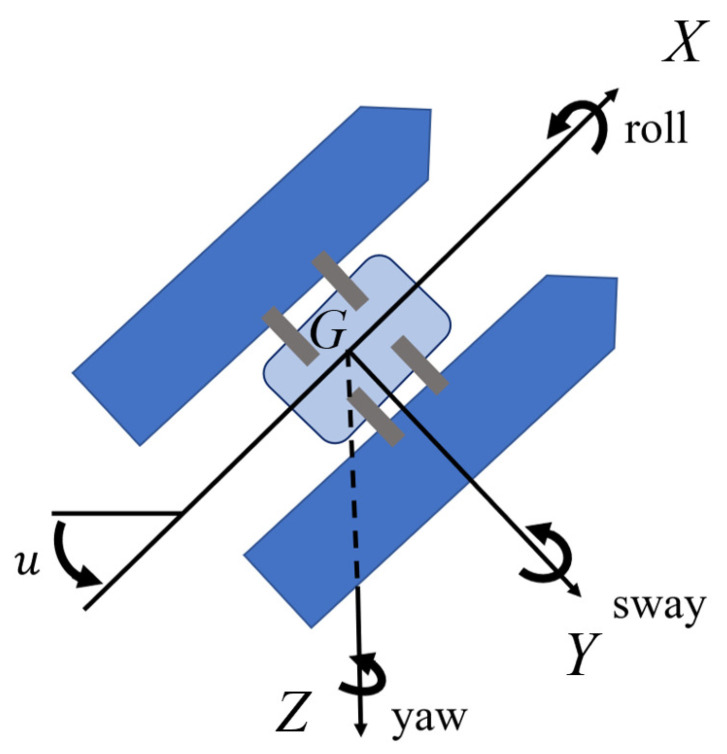
Motion coordinate system of USV.

By translating the equations in (1) to the coordinate system presented in Figure 1, where G in Figure 1 denotes the center of gravity, adopting Taylor expansion and Laplace transformation, and disregarding some hydrodynamic effects, one can achieve the following transfer functions for the models of the hydrodynamics forces:

$$\left\{\begin{array}{c} \theta \left(s\right)=\frac{X_{dv}}{1+T_{v}s}u\left(s\right)\\ \psi \left(s\right)=\frac{1}{1+T_{r}s}\left[X_{dr}u\left(s\right)+X_{vr}\theta \left(s\right)+W\left(s\right)\right]\\ \varphi \left(s\right)=\frac{\omega_{n}^{2}s}{s^{2}+2\omega_{n}𝜕s+\omega_{n}^{2}}\left[X_{dp}u\left(s\right)+X_{vp}\theta \left(s\right)+V\left(s\right)\right] \end{array}\right.$$

where θ(s), ψ(s), φ(s), and u(s) denote the Laplace transformation of θ(t), ψ(t), φ(t), and u(t), respectively; u(t) denotes the rudder angle, which can be seen as the input, controlled by control devices, θ(t), ψ(t), and φ(t) denote the sway velocity caused by the rudder motion alone, the heading angle, and the roll angle, respectively, W(s) and V(s) represent the disturbances of waves and winds on ψ(s) and φ(s) under complex sea conditions, 𝜕 is the damping ratio, $T_{v}$ and $T_{r}$ are the time constants, and μ is the natural frequency. $X_{dv}$, $X_{dr}$, $X_{vr}$, $X_{dp}$, and $X_{vp}$ denote the known gains of transfer functions, which are linearly related to the forward speed (determined by the propeller), and given in Section 4.

Applying the inverse Laplace transform, the physical model (1) can be transformed into the state-space model in continuous time. Furthermore, forward Euler formula is unitized to convert the continuous-time model into the following discrete-time model [20]:

(2) $$\left\{\begin{array}{c} S_{o+1}=B_{1}S_{o}+B_{2}\sum_{t=0}^{o}S_{t}+Cu_{o}+DW_{o},\\ Z_{o}=E\sum_{t=0}^{o}S_{t}+GV_{o}, \end{array}\right.$$

where $S_{o}={\left[\begin{array}{ccc} \theta_{o} & \psi_{o} & \varphi_{o} \end{array}\right]}^{T}\in R^{n}$ represents the discrete-time state vector and $Z_{o}={\left[\begin{array}{cc} \pi_{o} & \phi_{o} \end{array}\right]}^{T}\in R^{m}$ represents the measurement output in which $\pi_{o}$ and $\phi_{o}$ denote the heading angle and the roll angle, respectively. $W_{o}$ and $V_{o}$ are process noise and measurement noise, respectively. $u_{o}\in R^{n}$ denotes the rudder angle. Transition matrices $B_{1}$, $B_{2}$, *C*, *D*, *E*, and *G* are determined as:

$$\begin{array}{cc} & B_{1}=\left[\begin{array}{ccc} -\frac{\Delta t}{T_{v}}+1 & 0 & 0\\ \frac{\Delta t}{T_{r}}X_{vr} & -\frac{\Delta t}{T_{v}}+1 & 0\\ \mu^{2}\Delta tX_{vp} & 0 & -2𝜕\mu \Delta t+1 \end{array}\right],\\ & B_{2}=\mathrm{diag}\;\;\;\left\{\;\;\;0,0,-\mu^{2}{\left(\Delta t\right)}^{2}\;\;\;\right\}\;\;\;,\\ & C=\left[\begin{array}{c} \frac{\Delta t}{T_{v}}X_{dv}\\ \frac{\Delta t}{T_{r}}X_{dr}\\ \mu^{2}\Delta tX_{dp} \end{array}\right],\;D=\left[\begin{array}{cc} 0 & 0\\ \frac{\Delta t}{T_{r}} & 0\\ 0 & \mu^{2}\Delta t \end{array}\right],\\ & E=\left[\begin{array}{ccc} 0 & \Delta t & 0\\ 0 & 0 & \Delta t \end{array}\right]. \end{array}$$

**Assumption** **A1.** *In order to simulate the disturbances caused by waves and wind in real environments, this paper defines $W_{o}$ and $V_{o}$ as unknown but bounded noises, which satisfy the following ellipsoid sets:*

(3) $$\left\{\begin{array}{cc} \; & W_{o}^{T}R_{1,o}^{-1}W_{o}\leq 1,\\ \; & V_{o}^{T}R_{2,o}^{-1}V_{o}\leq 1, \end{array}\right.$$

*where $R_{1,o}$ and $R_{2,o}$ are known positive definite matrices.*

**Remark** **1.** *In most practical situations, noise in USV motion is often difficult to identify accurately and has persistent influences. For example, wave disturbance is an irregular random sequence influenced by factors such as water density, hull length, wind force and tide, with extremely complex properties. However, in different cases, researchers can roughly estimate the possible peaks and troughs of wave disturbances and thus use them as boundary conditions. These boundary conditions can be transformed into ellipsoidal sets.*

**Remark** **2.** *In fact, the framework proposed in this paper is applicable not only to UBB noise, but also to other cases with bounded noise (including non-Gaussian noise with bounded amplitude). For unbounded noise, only estimation approaches that deal with unbounded noise can be used, such as particle filtering.*

Next, the parameter uncertainties are considered in the state-space model (2), which is rewritten as:

(4) $$\begin{array}{cc} \; & S_{o+1}=\left(B_{1}+\Delta B_{1o}\right)S_{o}+\left(B_{2}+\Delta B_{2o}\right)\sum_{t=0}^{o}S_{t}\\ \; & \;\;\;\;+\left(C+\Delta C_{o}\right)u_{o}+DW_{o}, \end{array}$$

with

(5) $$\begin{array}{cc} \; & \Delta B_{1o}=MQ_{o}F_{1},\\ \; & \Delta B_{2o}=MQ_{o}F_{2},\\ \; & \Delta C_{o}=MQ_{o}F_{3}, \end{array}$$

where $M\in R^{n\times g}$, $F_{1}\in R^{h\times n}$, $F_{2}\in R^{h\times n}$, and $F_{3}\in R^{h\times 1}$ are known real constant matrices. $Q_{o}\in R^{g\times h}$ is a norm bounded uncertain matrix and satisfies:

(6) $$Q_{o}^{T}Q_{o}\leq I.$$

### 2.2. Binary Coding Scheme

**Figure 2:**
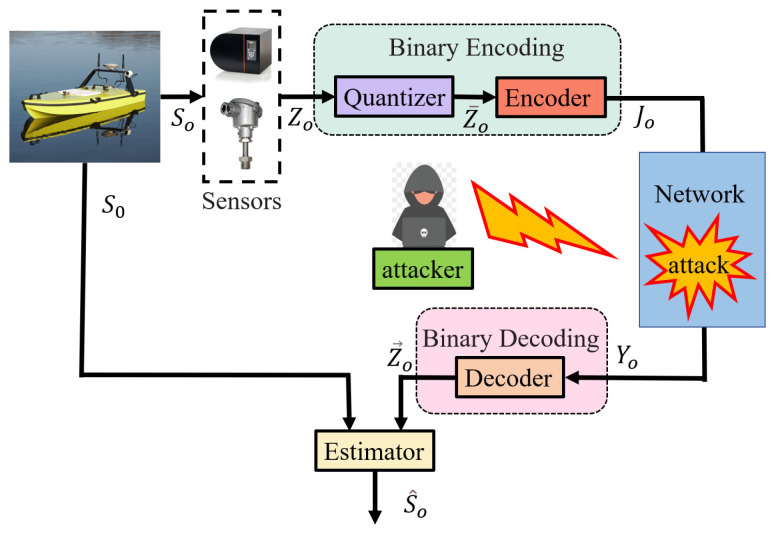
Structure of BCS.

Figure 2 illustrates the structure of the BCS. To accommodate the restricted bandwidth of the communication channel, the data sent by the sensors are initially quantized using a probability quantizer. Subsequently, the quantized data are converted into binary bit strings of finite length by the encoder. The binary bit strings are then transmitted to the decoder through a memoryless binary symmetric channels (BSC) and recovered by the decoder. Finally, the recovered data are forwarded to the estimator. The specific steps and formula derivations are outlined below:

Suppose $Z_{o}=\left[-\varpi ,\varpi \right]$, ϖ>0 need to be defined based on actual engineering conditions.

In traditional BCS, the length of the binary bit string is fixed. However, the operation environment of USV is complex and changeable. For example, when the steering motion data of USV are transmitted through the network channel, the network channel may need to transmit other data at the same time, which leads to part of the bandwidth being occupied. In this case, if we continue to transmit data in the original way, it may cause channel blockage, and then lead to the occurrence of time delays and missing measurements. Therefore, to solve this problem, we propose a dynamic selection strategy for the length of a binary bit string based on the original BCS. The introduction of this strategy can change the length of the binary bit string in real time, so as to effectively reduce the communication burden and alleviate the network blocking problem. The specific process of coding and decoding is as follows:

In order to encode the measured output $Z_{o}$ into binary strings with a growth degree of $p_{o}$, the interval [−ϖ,ϖ] needs to be divided into $2^{p_{o}}-1$ uniform intervals, each uniform interval with the following length:

(7) $$\kappa_{o}=\frac{2\varpi }{2^{p_{o}}-1}$$

In addition, the $2^{p_{o}}$ points defined as uniformly distributed are:

(8) $$N=\{n_{1},n_{2},\ldots ,n_{2^{p_{o}}}\},$$

where $n_{i}=-\varpi +\left(i-1\right)\kappa_{o}$, $\left(i=1,2,\ldots ,2^{p_{o}}\right)$.

A probability quantizer is used to pre-process the measurement data. When $n_{i}\leq Z_{o}\leq n_{i+1}$, the quantized measurement output ${\bar{Z}}_{o}$ is taken as follows:

(9) $$\begin{array}{cc} \; & \mathrm{Prob}\left\{{\bar{Z}}_{o}=n_{i}\right\}=1-\delta_{o},\\ \; & \mathrm{Prob}\left\{{\bar{Z}}_{o}=n_{i+1}\right\}=\delta_{o}, \end{array}$$

where $\delta_{o}=\frac{Z_{o}-n_{i}}{\kappa_{o}}$$\left(0\leq \delta_{o}\leq 1\right)$. Then, the quantization error is defined as follows:

(10) $$H_{1o}={\bar{Z}}_{o}-Z_{o}.$$

According to (9), random variable $H_{1o}$ satisfies:

(11) $$\begin{array}{cc} \; & P\left\{H_{1o}=-\delta_{o}\kappa_{o}\right\}=1-\delta_{o}\\ \; & P\left\{H_{1o}=\left(1-\delta_{o}\right)\kappa_{o}\right\}=\delta_{o} \end{array}$$

Further, utilizing the formula ${\bar{Z}}_{o}=-\varpi +\sum_{i=0}^{p_{o}}J_{i,o}2^{i-1}\kappa_{o}$, the encoder is able to convert the quantized measurement output into binary strings consisting of a set of bits:

$$J_{o}=\left\{J_{1,o},\;J_{2,o},\;\ldots ,\;J_{p_{o},o}\right\},\;J_{i,o}\in \left\{0,\;1\right\}$$.

Next, the encoder transmits binary strings to the decoder through memoryless BSC. It should be emphasized that during the transmission binary strings, the presence of complex surface weather conditions can introduce channel noises in the memoryless BSC, which can potentially cause each bit in the binary strings to flip with a certain probability. Therefore, the binary strings received by the decoder can be modeled as follows:

$$Y_{o}=\left\{Y_{1,o},Y_{2,o},\ldots ,Y_{p_{o},o}\right\},Y_{i,o}\in \left\{0,1\right\},$$

where $Y_{i,o}=\left(1-\lambda_{i,o}\right)J_{i,o}+\lambda_{i,o}\left(1-J_{i,o}\right)$ with

$$\lambda_{i,o}=\left\{\begin{array}{c} \begin{array}{cc} 1 & \mathrm{the}\;i\text{-}\mathrm{th}\;\mathrm{binary}\;\mathrm{bit}\;\mathrm{is}\;\mathrm{flipped}, \end{array}\\ \begin{array}{cc} 0 & \mathrm{the}\;i\text{-}\mathrm{th}\;\mathrm{binary}\;\mathrm{bit}\;\mathrm{is}\;\mathrm{not}\;\mathrm{flipped}. \end{array} \end{array}\right.$$

The random variable $\lambda_{i,o}$ satisfies:

(12) $$\begin{array}{cc} \; & P\{\lambda_{i,o}=1\}=\varepsilon ,\\ \; & P\{\lambda_{i,o}=0\}=1-\varepsilon , \end{array}$$

where ε∈[0,1] represents the flipping probability. For the sake of further analysis, we give the assumption that $\lambda_{i,o}$ are white and mutually independent. Subsequently, the decoder recovers the original signal using the following formula:

(13) $${\hat{Z}}_{o}=-\varpi +\sum_{i=0}^{p_{o}}Y_{i,o}2^{i-1}\kappa_{o}$$

**Lemma** **1** ([44]). *Assuming that the probability of flipping is ε during signal transmission via a memoryless BSC, then, the signal received by the decoder satisfies:*

$$\begin{array}{cc} & \left\{{\hat{Z}}_{o}\right\}=\left(1-2\varepsilon \right){\bar{Z}}_{o},\\ & V\left\{{\hat{Z}}_{o}\right\}=\varpi^{2}\chi , \end{array}$$

*where $\chi =\frac{4\varepsilon \left(1-\varepsilon \right)\left(2^{2p_{o}}-1\right)}{3{\left(2^{2p_{o}}-1\right)}^{2}}$.*

Considering Lemma 1, there is a certain degree of distortion between the signal ${\hat{Z}}_{o}$ recovered by the decoder and the signal $Z_{o}$ sent from the sensors. Therefore, the following formula is used to compensate for the distortion:

(14) $${\tilde{Z}}_{o}=\frac{1}{1-2\varepsilon }{\hat{Z}}_{o}.$$

The equivalent error, which is generated by signal transmission through memoryless BSC, is represented in the following form:

(15) $$H_{2o}={\tilde{Z}}_{o}-{\bar{Z}}_{o}.$$

The expectation and variance in $H_{2o}$ obtained from Lemma 1 are as follows:

(16) $$\begin{array}{cc} \; & \{H_{2o}\}=0,\\ \; & V\left\{H_{2o}\right\}=\frac{\varpi^{2}\chi }{{\left(1-2\varepsilon \right)}^{2}}. \end{array}$$

Combining the formulas (10) and (15), the recovered measurement output can be written as:

(17) $${\tilde{Z}}_{o}=Z_{o}+H_{1o}+H_{2o}.$$

Due to the open and unprotected nature of the networks, unauthorized individuals, such as illegal fishermen or competitors, can easily carry out cyber-attacks on the communication channels with the aim of sabotage. Moreover, in order to maximize the level of destruction, unauthorized individuals often combine multiple attack methods to launch mixed cyber-attacks against communication channels. To better reflect real scenarios, this paper assumes that communication channels are subjected to mixed cyber-attacks consisting of bias injection attack and DoS attack. As a result, the actual data outputted by the decoder are as follows:

(18) $${\vec{Z}}_{o}=\alpha_{o}\left({\tilde{Z}}_{o}+\beta_{o}\rho_{o}\right),$$

where $\alpha_{o}\begin{array}{c} \in \{0,1\} \end{array}$ and $\beta_{o}\begin{array}{c} \in \{0,1\} \end{array}$ represent the occurrence of DoS attack and bias injection attack, respectively. In engineering practice, due to the difficulty of a single DoS attack signal having a significant influence on the system, attackers often carry out continuous DoS attacks. Therefore, this paper pre-sets DoS attacks to occur continuously. $\alpha_{o}=0$ indicates that the channels are subjected to DoS attacks. $\alpha_{o}=1$ indicates that the channels are not subjected to DoS attacks. On the contrary, bias injection attacks occur stochastically and follow the Bernoulli distribution:

(19) $$\begin{array}{cc} \; & P\{\beta_{o}=1\}=\beta ,\\ \; & P\{\beta_{o}=0\}=1-\beta , \end{array}$$

where $\beta_{o}=1$ indicates that the channels are subjected to bias injection attacks. $\beta_{o}=0$ indicates that the channels are not subjected to bias injection attacks. $\rho_{o}$ represents an injection attack signal that meets the following conditions:

(20) $$E\{\rho_{o}^{T}\omega^{-1}\rho_{o}\}\;\leq 1.$$

### 2.3. Design Objective

Considering the mixed cyber-attacks, BCS, parameter uncertainties and estimator gain perturbations, the resilient set-membership estimator is designed as follows:

(21) $$\begin{array}{cc} \; & K_{f,o}=K_{o}+\Delta K_{o},\\ \; & {\hat{S}}_{o+1}=B_{1}{\hat{S}}_{o}+B_{2}\sum_{t=0}^{o}{\hat{S}}_{t}+Cu_{o}+K_{f,o}\left({\vec{Z}}_{o}-\alpha_{o}E\sum_{t=0}^{o}{\hat{S}}_{t}\right), \end{array}$$

where ${\hat{S}}_{o}\in R^{n}$ is the estimate of the vector $S_{o}$, $K_{o}$ is the gain matrix of estimator to be determined in the following content, and $\Delta K_{o}$ represents the estimator gain perturbation and satisfies:

(22) $$\Delta K_{o}=\hat{M}{\hat{Q}}_{o}\hat{F},$$

where ${\hat{Q}}_{o}$ is an unknown time-varying matrix satisfies ${\hat{Q}}_{o}^{T}{\hat{Q}}_{o}\leq I$, $\hat{M}$, and $\hat{F}$ are known real matrices of proper dimensions.

**Assumption** **2.** *When o=0, the estimate error is confined to the ellipsoidal region shown below:*

(23) $${\left(S_{o}-{\hat{S}}_{o}\right)}^{T}P_{o}^{-1}\left(S_{o}-{\hat{S}}_{o}\right)\leq 1,$$

*where $P_{o}$ is the positive define matrix.*

**Remark** **3.** *In practice, some uncertainty errors may be introduced in measuring the initial state of the system due to some unknown uncertainties (such as hydrodynamic effects and noises) in the initial state. However, by knowing the bounds of these parameters in advance, we can make specific assumptions about the state and thus treat these uncertainty errors as similar to the UBB noise described in Assumptions A1.*

This paper has two objectives. The first objective is to look for the estimator gain matrix $K_{j}\left(j=1,2,\ldots ,n\right)$, so that the $P_{j}$ and ${\hat{S}}_{j}$ are limited within the following ellipsoidal region:

(24) $${\left(S_{j}-{\hat{S}}_{j}\right)}^{T}P_{j}^{-1}\left(S_{j}-{\hat{S}}_{j}\right)\leq 1,$$

The second objective is to minimize the above ellipsoid in the sense of trace.

To facilitate subsequent derivation, at the end of this chapter, we introduce several lemmas:

**Lemma** **2** ([50]). *For constant matrices ${℘}_{1}$, ${℘}_{2}$, and ${℘}_{3}$ satisfying ${℘}_{1}={℘}_{1}^{T}$ and ${℘}_{2}={℘}_{2}^{T}>0$, then:*

$${℘}_{1}+{℘}_{3}^{T}{℘}_{2}^{-1}{℘}_{3}\leq 0.$$

*If and only if*

$$\left[\begin{array}{cc} {℘}_{1} & {℘}_{3}^{T}\\ {℘}_{3} & -{℘}_{2} \end{array}\right]\leq 0,\;\left[\begin{array}{cc} -{℘}_{2} & {℘}_{3}\\ {℘}_{3}^{T} & {℘}_{1} \end{array}\right]\leq 0,$$

**Lemma** **3** ([50]). *Let $z_{0}\left(\eta \right),\;z_{1}\left(\eta \right),\;\ldots ,\;z_{p}\left(\eta \right)$ be quadratic functions of $\eta \in R^{n}$: $z_{i}\left(\eta \right)=\eta H_{i}\eta$ with $H_{i}=H_{i}^{T}$.*

*Then, the following implication:*

$$z_{1}\left(\eta \right)\leq 0,\ldots ,\;z_{p}\left(\eta \right)\leq 0\Rightarrow z_{0}\left(\eta \right)\leq 0$$

*can be guaranteed if there are positive scalars ${ℵ}_{0},\;{ℵ}_{1},\;\ldots ,\;{ℵ}_{p}$ satisfying:*

$$H_{0}-\sum_{i=0}^{p}{ℵ}_{i}H_{i}\leq 0.$$

**Lemma** **4** ([50]). *For given matrices $\&={\&}^{T}$, ℜ and ε with appropriate dimensions, if and only if there is a positive scalar ϱ satisfying $+\varrho^{-1}ℜ{ℜ}^{T}+\varrho \varepsilon^{T}\varepsilon <0$ for all $M_{o}$ satisfying $M_{o}^{T}M_{o}\leq I$, the following inequality:*

$$\&+ℜM_{o}\varepsilon +\varepsilon^{T}M_{o}^{T}{ℜ}^{T}<0$$

*holds.*

## 3. Main Result

### 3.1. Design of Robust Set-Membership Estimator

In this section, we achieve the first objective by the subsequent theorem: obtaining sufficient conditions for the estimation errors to be limited within specified ellipsoidal regions at each transmission time.

**Theorem** **1.** *Considering the USV models (4), suppose positive-definite matrix $P_{0}$ is given, and limited in ellipsoid (23), the first objective can be achieved if there is a set of matrices $P_{o+1}$, $K_{o}$, scalar ζ and $\sigma_{i}$(i=1,2,…,7) such that:*

(25) $$\Upsilon =\left[\begin{array}{ccc} \tilde{\Upsilon } & \tilde{M} & \zeta {\tilde{F}}^{T}\\ {}* & -\zeta I & 0\\ {}* & * & -\zeta I \end{array}\right]\leq 0,$$

*where*

$$\begin{array}{cc} \; & \tilde{\Upsilon }=\left[\begin{array}{cc} P_{o+1} & \Phi_{o}\\ {}* & \Lambda_{o} \end{array}\right],\\ \; & \Lambda_{o}=diag\;\;\;\{\;\;\;-\sigma_{1}I,-\sigma_{2}I,-\sigma_{3}R_{1,o}^{-1},-\sigma_{4}R_{2,o}^{-1},-\sigma_{5}I,-\sigma_{6}I,\\ \; & \;\;\;\;\;-\sigma_{7}\omega^{-1},\Lambda_{2o}\;\;\;\}\;\;\;\\ \; & \Lambda_{2o}=-1+\sigma_{1}+o\sigma_{2}+\sigma_{3}+\sigma_{4}+m\kappa_{o}^{2}\sigma_{5}+m\frac{\varpi^{2}}{{\left(1-2\varepsilon \right)}^{2}}\sigma_{6}+\sigma_{7},\\ \; & \Phi_{o}=\left[\begin{array}{cc} {\Phi_{o}^{\left(1\right)}}^{T} & {{\tilde{\Phi }}_{o}^{\left(2\right)}}^{T} \end{array}\right],\\ \; & \Phi_{o}^{\left(1\right)}=[\Phi_{1o}\;\Phi_{2o}\;D\;-\left(\Delta K_{o}+K_{o}\right)\alpha_{o}G\;-\left(\Delta K_{o}+K_{o}\right)\alpha_{o}\;\\ \; & \;\;\;-\left(\Delta K_{o}+K_{o}\right)\alpha_{o}\;-\left(\Delta K_{o}+K_{o}\right)\alpha_{o}\bar{\beta }\;\Phi_{3o}],\\ \; & {\tilde{\Phi }}_{o}^{\left(2\right)}=\left[\begin{array}{cccccccc} 0 & 0 & 0 & 0 & 0 & 0 & -\left(K_{o}+\Delta K_{o}\right)\alpha_{o}\tilde{\beta } & 0 \end{array}\right],\\ \; & \tilde{\beta }=\sqrt{\bar{\beta }\left(1-\bar{\beta }\right)},\\ \; & \Phi_{1o}=\left(B_{1o}+\Delta B_{1o}\right)L_{o}, \end{array}$$

$$\begin{array}{cc} \; & \Phi_{2o}=\left(B_{2o}+\Delta B_{2o}-\left(K_{o}+\Delta K_{o}\right)\alpha_{o}E\right)L_{\tau o},\\ \; & \Phi_{3o}=\Delta B_{1o}{\hat{S}}_{o}+\Delta B_{2o}\sum_{t=0}^{o}{\hat{S}}_{t}+\Delta Cu_{o},\\ \; & L_{\tau o}=\left[\begin{array}{cccccc} L_{0} & L_{1} & L_{2} & L_{3} & \ldots & L_{o} \end{array}\right],\\ \; & \tilde{M}={\left[\begin{array}{cccc} \begin{array}{cc} {\tilde{M}}_{1}^{T} & 0 \end{array} & 0 & 0 & \begin{array}{ccc} \begin{array}{cc} \begin{array}{cc} 0 & 0 \end{array} & 0 \end{array} & 0 & 0 \end{array} \end{array}\right]}^{T},\\ \; & {\tilde{M}}_{1}=\left[\begin{array}{ccccc} M & M & M & \hat{M} & 0\\ 0 & 0 & 0 & 0 & \hat{M} \end{array}\right],\\ \; & \;\tilde{F}=\left[\begin{array}{cc} 0 & F \end{array}\right],\;F=\left[\begin{array}{cc} {\overset{⇀}{F}}_{1} & {\overset{⇀}{F}}_{2} \end{array}\right],\;\\ \; & {\overset{⇀}{F}}_{1}=\left[\begin{array}{cccc} F_{1}L_{o} & 0 & 0 & 0\\ 0 & F_{2}L_{\tau o} & 0 & 0\\ 0 & 0 & 0 & 0\\ 0 & -\hat{F}\alpha_{o}EL_{\tau o} & 0 & -\hat{F}\alpha_{o}G\\ 0 & 0 & 0 & 0 \end{array}\right],\\ \; & {\overset{⇀}{F}}_{2}=\left[\begin{array}{cccc} 0 & 0 & 0 & F_{1}{\hat{S}}_{o}\\ 0 & 0 & 0 & F_{2}\sum_{t=0}^{o}{\hat{S}}_{t}\\ 0 & 0 & 0 & F_{3}u_{o}\\ -\hat{F}\alpha_{o} & -\hat{F}\alpha_{o} & -\hat{F}\alpha_{o}\bar{\beta } & 0\\ 0 & 0 & -\hat{F}\alpha_{o}\tilde{\beta } & 0 \end{array}\right], \end{array}$$

*in which $L_{o}$ is the Cholesky factorization of $P_{o}=L_{o}^{T}L_{o}$.*

**Proof.**  According to Assumption 2, we know ${\left(S_{0}-{\hat{S}}_{0}\right)}^{T}$$P_{0}^{-1}\left(S_{0}-{\hat{S}}_{0}\right)\leq 1$. Next, we assume that ${\left(S_{i}-{\hat{S}}_{i}\right)}^{T}P_{i}^{-1}\left(S_{i}-{\hat{S}}_{i}\right)\leq 1$, i∈{1,2,…,o}. Finally, by using mathematical induction, we need to prove that ${\left(S_{i+1}-{\hat{S}}_{i+1}\right)}^{T}P_{i+1}^{-1}\left(S_{i+1}-{\hat{S}}_{i+1}\right)\leq 1$, ensuring that the estimation errors are always confined within specific ellipsoidal regions.

To begin with, we calculate the estimation error and express it in the following form:

(26) $$\begin{array}{cc} \; & e_{o+1}=S_{o+1}-{\hat{S}}_{o+1}\\ \; & \;\;\;=\left(\Delta B_{2o}+B_{2o}-\left(\Delta K_{o}+K_{o}\right)\alpha_{o}E\right)\sum_{t=0}^{o}e_{t}\\ \; & \;\;\;\;\;-\left(\Delta K_{o}+K_{o}\right)\alpha_{o}GV_{o}+\Delta C_{o}u_{o}\\ \; & \;\;\;\;\;+\left(\Delta B_{1o}+B_{1o}\right)e_{o}+DW_{o}\\ \; & \;\;\;\;\;-\left(\Delta K_{o}+K_{o}\right)\alpha_{o}H_{1o}\\ \; & \;\;\;\;\;-\left(\Delta K_{o}+K_{o}\right)\alpha_{o}H_{2o}\\ \; & \;\;\;\;\;-\left(\Delta K_{o}+K_{o}\right)\alpha_{o}\beta_{o}\rho_{o}\\ \; & \;\;\;\;\;-\Delta B_{1o}{\hat{S}}_{o}-\Delta B_{2o}\sum_{t=0}^{o}{\hat{S}}_{t}. \end{array}$$

On the basis of [51], there is a vector $\mu_{o}$ satisfying $\mu_{o}^{T}\mu_{o}\leq I$ such that

$$S_{o}-{\hat{S}}_{o}=L_{o}\mu_{o}.$$

Therefore, we rewrite the estimation error as follows:

(27) $$e_{o+1}=S_{o+1}-{\hat{S}}_{o+1}=\Phi_{o}\xi_{o},$$

where

$$\begin{array}{cc} & \Phi_{o}=(\Phi_{1o}\;\Phi_{2o}\;D\;-\left(\Delta K_{o}+K_{o}\right)\alpha_{o}G\;-\left(\Delta K_{o}+K_{o}\right)\alpha_{o}\;\\ & \;\;\;-\left(\Delta K_{o}+K_{o}\right)\alpha_{o}\;-\left(\Delta K_{o}+K_{o}\right)\alpha_{o}\beta_{o}\;\Phi_{3o}),\\ & \xi_{o}={\left(\begin{array}{cccccccc} \mu_{o}^{T} & \mu_{\tau o}^{T} & W_{o}^{T} & V_{o}^{T} & H_{1o}^{T} & H_{2o}^{T} & \rho_{o}^{T} & I^{T} \end{array}\right)}^{T},\\ & \mu_{\tau o}={\left(\begin{array}{cccccc} \mu_{0}^{T} & \mu_{1}^{T} & \mu_{2}^{T} & \mu_{3}^{T} & \ldots & \mu_{o}^{T} \end{array}\right)}^{T}, \end{array}$$

where $\Phi_{1o}$, $\Phi_{2o}$, and $\Phi_{3o}$ are given in (25).

Next, we can decompose the stochastic matrix $\Phi_{o}$ as follows:

(28) $$\Phi_{o}=\Phi_{o}^{\left(1\right)}+{\tilde{\Phi }}_{o}^{\left(2\right)},$$

where

$$\begin{array}{cc} & {\tilde{\Phi }}_{o}^{\left(2\right)}=\left(\begin{array}{cccccccc} 0 & 0 & 0 & 0 & 0 & 0 & -\left(K_{o}+\Delta K_{o}\right)\alpha_{o}{\overset{⌣}{\beta }}_{o} & 0 \end{array}\right),\\ & {\overset{⌣}{\beta }}_{o}=\beta_{o}-\bar{\beta }. \end{array}$$

Then, we can acquire:

(29) $$\begin{array}{cc} \; & \;E\;\;\;\{\;\;\;{\left(S_{o}-{\hat{S}}_{o}\right)}^{T}P_{o+1}^{-1}\left(S_{o}-{\hat{S}}_{o}\right)\;\;\;\}\;\;\;\\ \; & =E\;\;\;\{\;\;\;\xi_{o}^{T}\Phi_{o}^{T}P_{o+1}^{-1}\Phi_{o}\xi_{o}\;\;\;\}\;\;\;\\ \; & =\xi_{o}^{T}({\Phi_{o}^{\left(1\right)}}^{T}P_{o+1}^{-1}\begin{array}{cc} \Phi_{o}^{\left(1\right)}+{{\tilde{\Phi }}_{o}^{\left(2\right)}}^{T}P_{o+1}^{-1}{\tilde{\Phi }}_{o}^{\left(2\right)})\xi_{o} & \end{array}\\ \; & =\xi_{o}^{T}\Psi_{o}\xi_{o}. \end{array}$$

From (3), (11), (16), (20), and (27), we can acquire:

(30) $$\begin{array}{cc} \; & W_{o}^{T}R_{1,o}^{-1}W_{o}\leq 1,\;V_{o}^{T}R_{2,o}^{-1}V_{o}\leq 1,\\ \; & \rho_{o}^{T}\omega^{-1}\rho_{o}\leq 1,\;H_{1o}^{T}H_{1o}\leq m\kappa_{o}^{2},\\ \; & H_{2o}^{T}H_{2o}\leq m\frac{\varpi^{2}}{{\left(1-2\varepsilon \right)}^{2}},\\ \; & \mu_{o}^{T}\mu_{o}\leq 1,\;\mu_{\tau o}^{T}\mu_{\tau o}\leq o, \end{array}$$

which can be rewritten with $\xi_{o}$ as follows:

(31) $$\left\{\begin{array}{c} \xi_{o}^{T}\mathrm{diag}\left\{I,0,0,0,0,0,0,-1\right\}\xi_{o}\leq 0,\\ \xi_{o}^{T}\mathrm{diag}\left\{0,I,0,0,0,0,0,-o\right\}\xi_{o}\leq 0,\\ \xi_{o}^{T}\mathrm{diag}\left\{0,0,R_{1,o}^{-1},0,0,0,0,-1\right\}\xi_{o}\leq 0,\\ \xi_{o}^{T}\mathrm{diag}\left\{0,0,0,R_{2,o}^{-1},0,0,0,-1\right\}\xi_{o}\leq 0,\\ \xi_{o}^{T}\mathrm{diag}\left\{0,0,0,0,I,0,0,-m\kappa_{o}^{2}\right\}\xi_{o}\leq 0,\\ \xi_{o}^{T}\mathrm{diag}\left\{0,0,0,0,0,I,0,-m\frac{\varpi^{2}}{{\left(1-2\varepsilon \right)}^{2}}\right\}\xi_{o}\leq 0,\\ \xi_{o}^{T}\mathrm{diag}\left\{0,0,0,0,0,0,\omega^{-1},-1\right\}\xi_{o}\leq 0. \end{array}\right.$$

According to Lemma 3, if there is a set of positive scalars $\sigma_{i}$(i=1,2,…,7) that:

(32) $$\begin{array}{cc} \; & \Psi_{o}-\sigma_{1}\mathrm{diag}\left\{I,0,0,0,0,0,0,-1\right\}\\ \; & -\sigma_{2}\mathrm{diag}\left\{0,I,0,0,0,0,0,-o\right\}\\ \; & -\sigma_{3}\mathrm{diag}\left\{0,0,R_{1,o}^{-1},0,0,0,0,-1\right\}\\ \; & -\sigma_{4}\mathrm{diag}\left\{0,0,0,R_{2,o}^{-1},0,0,0,-1\right\}\\ \; & -\sigma_{5}\mathrm{diag}\left\{0,0,0,0,I,0,0,-m\kappa_{o}^{2}\right\}\\ \; & -\sigma_{6}\mathrm{diag}\left\{0,0,0,0,0,I,0,-m\frac{\varpi^{2}}{{\left(1-2\varepsilon \right)}^{2}}\right\}\\ \; & -\sigma_{7}\mathrm{diag}\left\{0,0,0,0,0,0,\omega^{-1},-1\right\}\\ \; & -\mathrm{diag}\{0,0,0,0,0,0,0,1\}\leq 0, \end{array}$$

Then, the inequality $\xi_{o}^{T}\left(\Psi_{o}-\mathrm{diag}\left\{0,0,0,0,0,0,0,-1\right\}\right)\xi_{o}\leq 0$ holds and (32) can be expressed in an alternative form:

(33) $$\Psi_{o}-\Lambda_{o}\leq 0,$$

where $\Lambda_{o}$ is given in (25).

Based on Lemma 2, we can deduce that (33) is equal to LMI as follows:

(34) $$\tilde{\Upsilon }=\left[\begin{array}{cc} -P_{o+1} & \Phi_{o}\\ {}* & \Lambda_{o} \end{array}\right]\leq 0.$$

This also means that, without considering parameter uncertainty and estimator gain perturbation, the estimation error can be limited within a specified ellipsoidal region at each transmission time.

The following content is used to establish the framework of resilient estimation, which can handle the influence caused by parameter uncertainties and estimator gain perturbations. Due to $\Phi_{o}={\overset{↼}{\Phi }}_{o}+{\overset{⇀}{\Phi }}_{o},\tilde{\Upsilon }$ can be decomposed by:

(35) $$\left[\begin{array}{cc} -P_{o+1} & \Phi_{o}\\ {}* & \Lambda_{o} \end{array}\right]=\left[\begin{array}{cc} -P_{o+1} & {\overset{↼}{\Phi }}_{o}\\ {}* & \Lambda_{o} \end{array}\right]+\left[\begin{array}{cc} 0 & {\overset{⇀}{\Phi }}_{o}\\ {}* & 0 \end{array}\right],$$

where

$$\begin{array}{cc} & {\overset{⇀}{\Phi }}_{o}=\left[\begin{array}{cc} {{\overset{⇀}{\Phi }}_{o}^{\left(1\right)}}^{T} & {{\tilde{\overset{⇀}{\Phi }}}_{o}^{\left(2\right)}}^{T} \end{array}\right],\\ & {\overset{⇀}{\Phi }}_{o}^{\left(1\right)}=(\Delta B_{1o}L_{o}\;{\overset{⇀}{\Phi }}_{2o}\;0\;-\Delta K_{o}\alpha_{o}G\;\\ & \;\;\;\;-\Delta K_{o}\alpha_{o}\;-\Delta K_{o}\alpha_{o}\;-\Delta K_{o}\alpha_{o}\bar{\beta }\;{\overset{⇀}{\Phi }}_{3o}),\\ & {\overset{⇀}{\Phi }}_{2o}=\left(\Delta B_{2o}-\Delta K_{o}\alpha_{o}E\right)L_{\tau o},\\ & {\overset{⇀}{\Phi }}_{3o}=\Delta B_{1o}{\hat{S}}_{o}+\Delta B_{2o}\sum_{t=0}^{o}{\hat{S}}_{t}+\Delta Cu_{o},\\ & {\tilde{\overset{⇀}{\Phi }}}_{o}^{\left(2\right)}=\left[\begin{array}{cccccccc} 0 & 0 & 0 & 0 & 0 & 0 & -\Delta K_{o}\alpha_{o}\tilde{\beta } & 0 \end{array}\right]. \end{array}$$

Then, combining (5) and (22), we can obtain that:

(36) $$\tilde{\Upsilon }+\tilde{M}{\tilde{Q}}_{o}\tilde{F}+{\tilde{F}}^{T}{\tilde{Q}}_{o}^{T}{\tilde{M}}^{T}<0,$$

where $\tilde{M}$ and $\tilde{F}$ are given in (25) and ${\tilde{Q}}_{o}=\mathrm{diag}\left\{Q_{o},Q_{o},Q_{o},{\hat{Q}}_{o},{\hat{Q}}_{o}\right\}$. According to Lemma 4, there is a positive scalar ζ such that:

(37) $$\tilde{\Upsilon }+\;\zeta^{-1}\tilde{M}{\tilde{M}}^{T}+\zeta {\tilde{F}}^{T}\tilde{F}<0,$$

Then, according to Lemma 4, it can be deduced that the inequality (37) is equivalent to (25), and the proof of Theorem 1 is completed.    □

### 3.2. Optimization Problem

By solving the set of aforementioned LMIs iteratively, the estimator gain (if it exists) can be obtained. However, the resulting estimator gain may be a set rather than a single value. To obtain the optimal estimator gain within this set, in the sense of trace, we minimize the ellipsoidal constraint $P_{o}$. Corollary 1. If there are non-negative scalars $\sigma_{i}$(i=1, 2 ,…, 7), ζ, and a set of matrices $K_{o}$, such that the following optimization problem is solvable:

**Corollary** **1.** *If there are non-negative scalars $\sigma_{i}$(i=1,2,…,7), ζ, and a set of matrices $K_{o}$ such that the following optimization problem is solvable:*

(38) $$\min_{P_{o+1}>0,\;K_{o},\;\sigma_{i}\left(i=1,\;2,\;\ldots ,\;7\right),\;\zeta }\;\left(\mathrm{trace}(P_{o+1})\right)\;\mathrm{if}\;(25)\;\mathrm{holds}$$

*then a minimized ellipsoidal region (in the sense of trace) is guaranteed, and an optimal estimator gain can be obtained at each transmission time.*

The pseudocode for proposed estimation framework is shown in Algorithm 1.
**Algorithm** **1:** Set-membership estimation framework for USV steering motion**Input Data:** Initial motion state $S_{0}$, initial motion estimation ${\hat{S}}_{0}$, initial ellipsoid       parameters $P_{0}$, total simulation time $o_{\max}$, rudder angle $u_{0}$, the upper and       lower limits of quantification ±ϖ.**Output:** Estimation value ${\hat{S}}_{i}$$\left(i\in \left[1,o_{\max}\right]\right)$ of motion state.Generate $S_{0}$, ${\hat{S}}_{0}$, $P_{0}$, $o_{\max}$, $u_{0}$, ±ϖ;       for $o=1:o_{\max}$   **step 1:** Generate process noise $W_{o}$;   **step 2:** Generate measurement noise $V_{o}$;   **step 3:** Calculate real motion state $S_{o}$ and its measurement $Z_{o}$;   **step 4:** Select the length of a binary bit string $p_{o}$;   **step 5:** Perform the binary coding for $Z_{o}$, which includes quantization,       encoding, transmission and decoding;   **step 6:** Calculate decoded measurement ${\vec{Z}}_{o}$;   **step 7:** Acquire the optimal estimator gain $K_{o}$ by solving optimization       problem Corollary 1 subjected to LMIs (25);   **step 8:** Calculate the estimation value ${\hat{S}}_{o+1}$ based on ${\hat{S}}_{o}$, $K_{o}$, and ${\vec{Z}}_{o}$.  end

**Figure 3:**
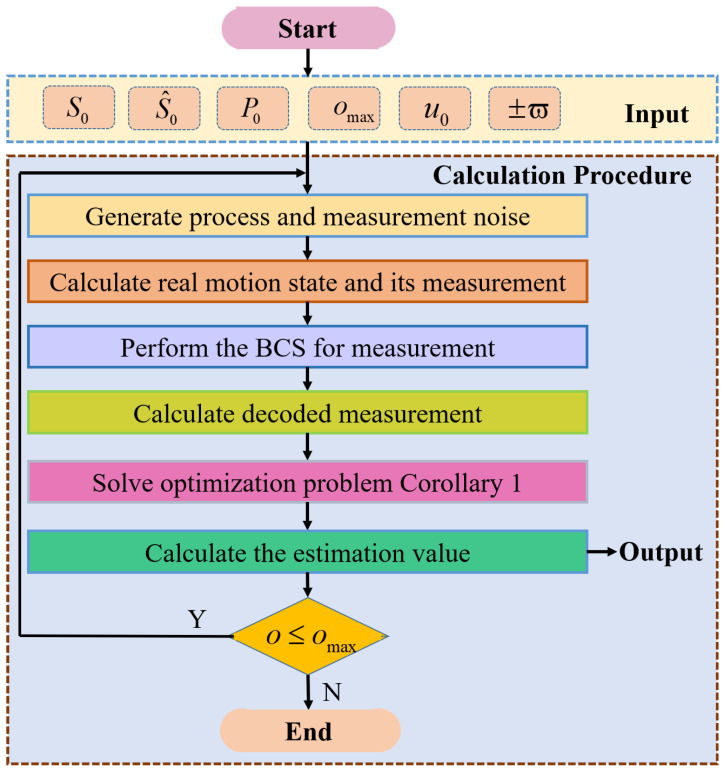
Flow chart of proposed estimation framework.

The flow chart for proposed estimation framework is shown in Figure 3.

## 4. Simulation Experiment

### 4.1. Numerical Simulation

According to (1), in the USV steering motion, the values of various parameters are obtained from [20] and shown below:

$$\begin{array}{cc} & \omega_{n}=2.3\;\left(\mathrm{rad}/s\right),\;X_{vp}=0.25S,\;X_{dp}=0.003S^{2}\\ & X_{vr}=-0.58\;\left(m/s\right),\;X_{dv}=-0.018S,\;X_{dv}=0.01S\\ & T_{r}=2/S,\;T_{v}=1.8/S,\;S=5\;\left(m/s\right),\;𝜕=0.48+0.15S\\ & \Delta t=0.1\;s,\;o_{\max}=85, \end{array}$$

where Δt is the sampling interval, *S* is the constant forward speed, and $o_{\max}$ is the maximum transmission time. Therefore, the state transition matrices $B_{1}$, $B_{2}$, *C*, *D*, *E*, and *G* are calculated as:

$$\begin{array}{cc} & B_{1}=\left[\begin{array}{ccc} 0.7222 & 0 & 0\\ -0.145 & 0.75 & 0\\ 0.6612 & 0 & 0.4342 \end{array}\right],\;C=\left[\begin{array}{c} 0.0139\\ -0.0225\\ -0.0397 \end{array}\right],\\ & B_{2}=\mathrm{diag}\left\{0,0,-0.0529\right\},\;E=\left[\begin{array}{ccc} 0 & 0.1 & 0\\ 0 & 0 & 0.1 \end{array}\right],\\ & G=\left[\begin{array}{c} 0.7\\ 0.8 \end{array}\right],\;D=\left[\begin{array}{cc} 0 & 0\\ 0.25 & 0\\ 0 & 0.529 \end{array}\right]. \end{array}$$

The parameter uncertainties are defined as:

$$\begin{array}{cc} & F_{1}=\left[\begin{array}{ccc} -0.01 & 0.01 & 0.05 \end{array}\right],\;F_{2}=\left[\begin{array}{ccc} 0.02 & 0.03 & -0.02 \end{array}\right]\\ & F_{3}=0.01,\;Q_{o}=\sin(rand\left(1\right)*2\pi ),\;M={\left[\begin{array}{ccc} 0.01 & -0.02 & 0.01 \end{array}\right]}^{T}. \end{array}$$

The estimator gain perturbations are defined as:

$$\begin{array}{cc} & \hat{F}=\left[\begin{array}{ccc} -0.15 & 0.05 & 0.1 \end{array}\right],\;{\hat{Q}}_{o}=\sin(rand\left(1\right)*2\pi ),\;\hat{M}={\left[\begin{array}{cc} 0.1 & -0.05 \end{array}\right]}^{T}. \end{array}$$

The initial values are defined as:

$$\begin{array}{cc} & S_{0}={\left[\begin{array}{ccc} 0 & 0 & 0 \end{array}\right]}^{T},\;{\hat{S}}_{0}={\left[\begin{array}{ccc} 0.3 & 0.3 & 0.3 \end{array}\right]}^{T},\\ & P_{0}=\mathrm{diag}\left\{0.09,\;0.09,\;0.09\right\}. \end{array}$$

The parameters of process noises and measurement noises are defined as $W_{o}=0.1\sin(rand\left(1\right)*2\pi )$, $V_{o}=0.3\sin(rand\left(1\right)*2\pi )$, $R_{1,o}=0.01$, and $R_{2,o}=0.09$. The parameters of mixed cyber-attacks are defined as $\bar{\beta }=0.05$, $\rho_{o}=2\sin\left(o\right)$, ω=4, and $\alpha_{i}=0$(i=30,31,32,33,40,41,42,43). The parameters of BCS are defined as ϖ=0.4, ε=0.01. The rudder angle is defined as $u_{o}=4sin\left(0.8o\right)$.

**Remark** **4.** *In our study for the USV, the parameter selection involves the key factors such as the rudder angle, speed, and noise. The selection of these parameters should be appropriately adjusted according to the context of the research problem, such as the role, working environment, and performance characteristics of the USV. The relevant literature and corresponding sea condition information should be reviewed to determine the best parameter setting for different situations. For the selection of estimator gain, as discussed in the previous section, the estimator gain obtained by solving LMIs (25) may be a set. Therefore, in this paper, we need to solve an optimization problem (Corollary 1) that can obtain the local optimal estimator gain.*

MATLAB R2020a is used to calculate LMIs in Theorem 1, and the optimization problem (38) can be solved by the MATLAB function (sedumi).

**Case** **1.** In the following, we consider the case which DoS attack and bias injection attack occurs during the estimation process, and we compare the proposed estimation framework with the traditional set-membership estimation framework [52].

In case 1, the length of the binary bit string is set to $p_{o}=6\;\left(o\in \left[0,85\right]\right)$ because the network bandwidth is not occupied by other data.

**Figure 4:**
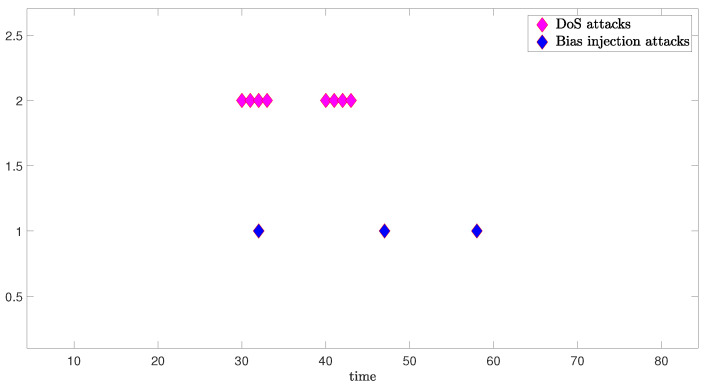
Occurrences of bias injection attacks and DoS attacks.

The schematic diagram which illustrates the occurrence of mixed cyber-attacks and the simulation results from three degrees of freedom are shown as follows:

Figure 4 illustrates the occurrence of mixed cyber-attacks. Considering that DoS attacks in real scenarios occur continuously, we pre-set the occurrence of the DoS attacks, and simulate the occurrence of bias injection attacks according to Bernoulli sequence distribution.

**Figure 5:**
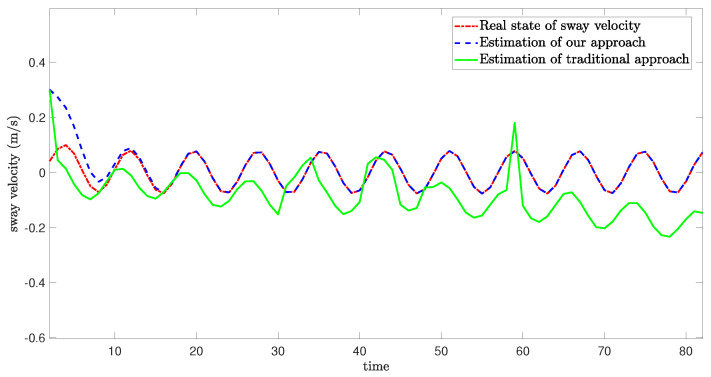
Real state of sway velocity and its estimation value.

**Figure 6:**
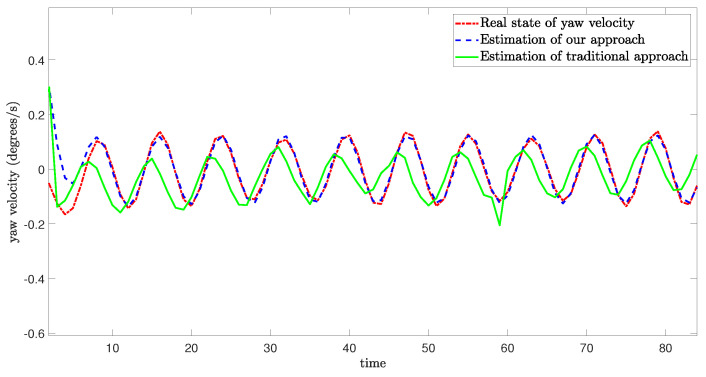
Real state of yaw velocity and its estimation value.

**Figure 7:**
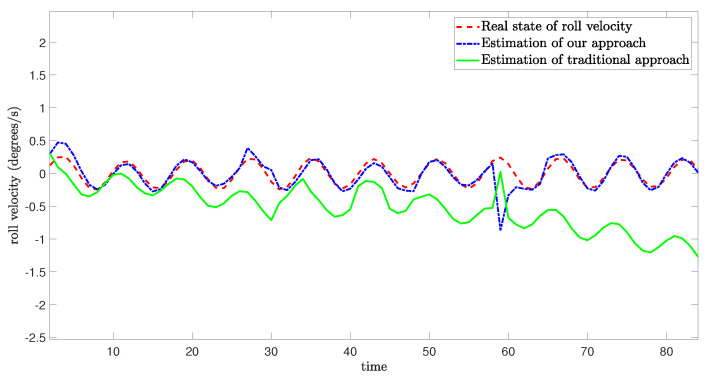
Real state of roll velocity and its estimation value.

Figure 5, Figure 6 and Figure 7 demonstrate the real state of the sway velocity, yaw velocity, and roll velocity, and their estimation values. From Figure 5, Figure 6 and Figure 7, we can see that although there are some influencing factors in the system, such as UBB noises, mixed cyber-attacks, model parameter uncertainties, estimator gain perturbations, and significant initial differences between the real state value and its estimation value. Moreover, the data in the channels need to be quantitated, encoded, and transmitted, which may cause certain errors. The improved resilient set-membership estimation framework is still capable of accurately estimate the sway velocity and yaw velocity. For the roll velocity, although the mixed cyber-attacks can have certain influences on the estimation performance, the improved resilient set-membership estimation framework can restore the estimation performance in a short time after the attack disappears.

Figure 5 and Figure 6 show that our proposed framework has good estimation performance. This is because according to [20], due to the unique dynamics and constraints of USV, as long as the algorithm is designed properly, noises and disturbances cannot have a greater influence on the sway velocity and yaw velocity estimation performance of USV. And combing with [43], we can see that the effective combination of BCS and set-membership filtering can give full play to their respective characteristics. Therefore, the influence of various errors caused by cyber-attacks and BCS on the estimation performance is reduced effectively.

In contrast, there is a large lag and deviation between the true state and the estimated value obtained by the traditional set-membership estimation framework under the same parameters, it should be noted that, due to the lack of BCS during data transmission, the estimation performance of the traditional set-membership estimation framework is easily influenced by mixed cyber-attacks, the emergence of attacks can lead to an increasing deviation between estimated values and true values under the traditional set-membership estimation framework, and the deviation not decrease with the disappearance of attacks. More importantly, in the actual transmission process, transmitting data directly without quantization can increase the communication burden, leading to packet loss, missing measurement, which can greatly reduce the estimation performance.

**Figure 8:**
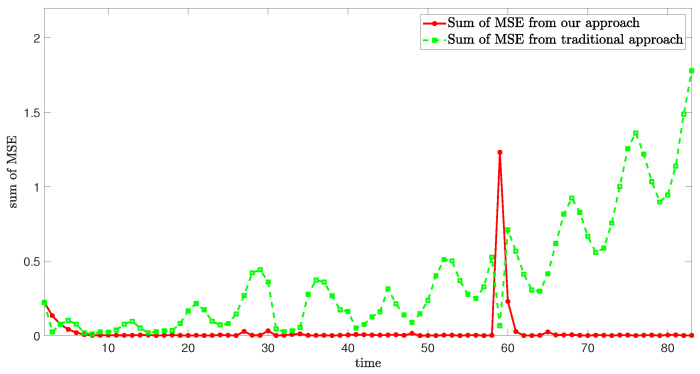
Sum of MSE from different approaches.

Figure 8 demonstrates the sum of mean square error (MSE) in case 1, which effectively denotes the high estimation accuracy of our approach.

**Case2.** For the complex operation environment of USV, we assume that part of the channel bandwidth is occupied during the time periods of [0,20] and [40,85]. Therefore, we need to adjust the binary bit string length of the data transmission in time to adapt to the changes in the channel bandwidth during the estimation process.

**Figure 9:**
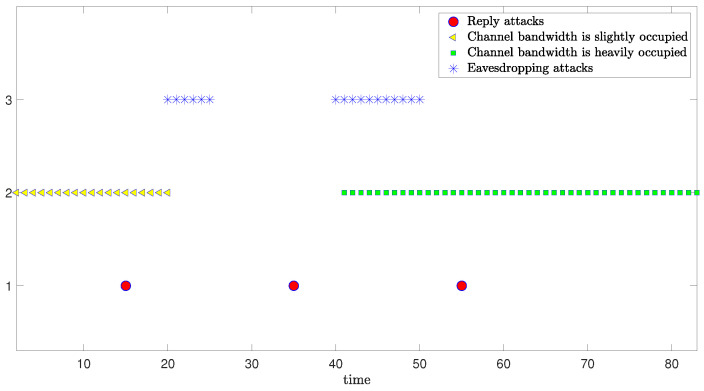
Occurrences of attacks and occupation of channel bandwidth.

**Figure 10:**
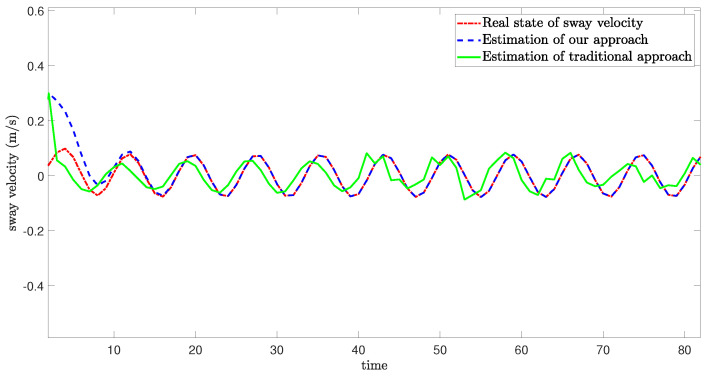
Real state of sway velocity and its estimation value.

**Figure 11:**
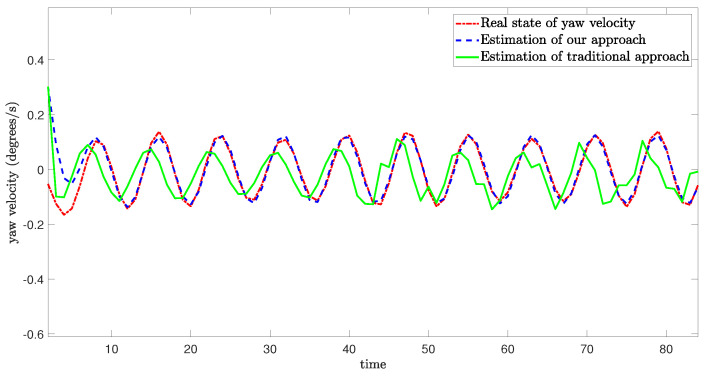
Real state of yaw velocity and its estimation value.

**Figure 12:**
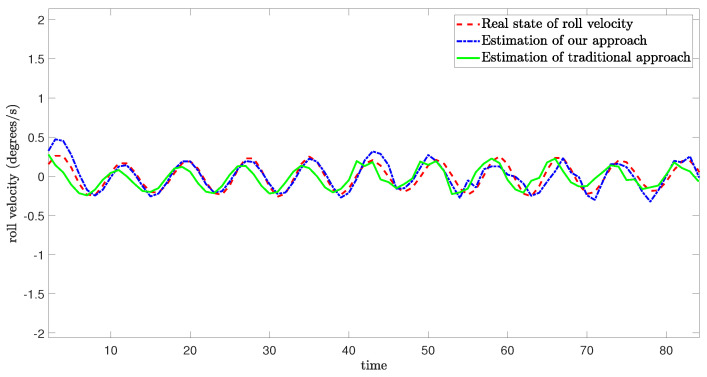
Real state of roll velocity and its estimation value.

**Figure 13:**
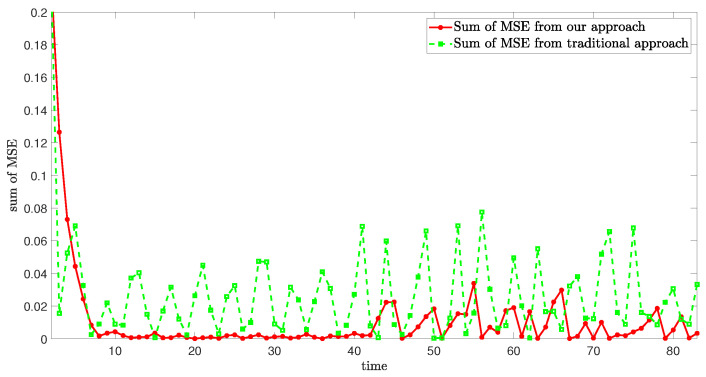
Sum of MSE from different approaches.

In addition, in order to comprehensively evaluate the effectiveness of our approach, in addition to the DoS attack and bias injection attack mentioned above, we also consider some other common types of cyber-attacks, such as replay attack and eavesdropping attack. Since the occurrence of replay attacks is difficult to describe by Bernoulli distribution [27], we therefore set those occurrences in advance to: ${\tilde{Z}}_{15}={\tilde{Z}}_{10}$, ${\tilde{Z}}_{35}={\tilde{Z}}_{28}$, ${\tilde{Z}}_{55}={\tilde{Z}}_{50}$, eavesdropping attacks are constant. The occurrence of cyber-attacks and occupation of channel bandwidth are shown in Figure 9:

For this complex situation, we compare our approach with traditional set-membership estimation framework under BCS with a dynamically selected binary bit string length strategy, after knowing that the channel bandwidth is occupied, we adjust the binary bit string length accordingly, specifically $p_{o}=4\;\left(o\in \left[0,20\right]\right)$, $p_{o}=6\;\left(o\in \left[21,40\right]\right)$, $p_{o}=2\;\left(o\in \left[41,85\right]\right)$, and the simulation results are as follows:

Figure 10, Figure 11 and Figure 12 demonstrate the real state of the sway velocity, yaw velocity, and roll velocity, and their estimation values. Figure 13 demonstrates the sum of MSE in case 2. From Figure 9, Figure 10, Figure 11, Figure 12 and Figure 13, we can see that as the length of the transmitted data decreases, the quantization error of the data also increases accordingly, and the estimation accuracy of the traditional approach is also greatly influenced. In contrast, our approach can reduce the influence of quantization error on the estimation accuracy, so as to effectively estimate the steering motion state of the USV. Moreover, our approach can effectively resist the influence of reply attacks on estimation performance. Finally, it should be noted that although the occurrence of eavesdropping attacks cannot be avoided, the data are encoded in binary before entering the network, and the principle of BCS is similar to encryption and decryption, and the upper and lower limits of quantification are the keys. Therefore, if the attackers do not know the specific key, they can only steal the binary array from the network, but cannot obtain the real value.

### 4.2. The Analysis of Experimental Results

In order to verify the estimation performance of the proposed estimation framework, we compare and calculate the computational load and MSE of the proposed approach and the traditional approach. The comparative estimation results of Case 1 are shown in Table 1 below.

According to Table 1, the MSE of the sway velocity, yaw velocity, and roll velocity of the traditional approach are higher than that of our approach by 93.00%, 73.32%, and 93.95%, respectively, and the calculated load of the traditional approach is higher than that of our approach by 4.38%.

The comparative estimation results of Case 2 are shown in Table 2 below.

According to Table 2, the MSE of the sway velocity, yaw velocity, and roll velocity of the traditional approach are higher than that of our approach by 44.76%, 73.64%, and 61.35%, respectively, and the calculated load of the traditional approach is higher than that of our approach by 5.01%. Therefore, the comprehensive comparison shows that our approach is optimal for the USV steering motion state estimation.

## 5. Conclusions

This paper has proposed a resilient set-membership state estimation algorithm based on BCS for the steering motion of USV under unknown but bounded noises. During the estimation process, the data of USV steering motion has been transmitted to the estimator by sensors via communication channels. These channels were bandwidth-constrained and subjected to cyber-attacks. Therefore, the BCS has been applied in communication channels, which not only saves communication resources but also ensures data quality while reducing the security risks caused by cyber-attacks. In addition, in order to simulate real sea conditions, this paper has considered the problems of parameter uncertainties and estimator gain perturbations. Thus, a resilient estimation framework has been designed to handle those problems. In the design process, the sufficient conditions for the existence of a resilient set-membership estimator have been established via the use of convex optimization method and mathematical induction method, which have ensured that the estimation error at each transmission time has been limited within a certain ellipsoidal region. Meanwhile, the estimator gain has been derived by iterative solution of a sequence of LMIs. Finally, the effectiveness of the established framework has been verified through a numerical simulation example.

There is a gap between theory and practice in our proposed estimation framework. Therefore, in our future research, we will take the following measures to bridge this gap, and apply estimation framework to practical projects after the theory is mature: (1) continuously calibrate the physical model of the system through a large amount of experimental data to ensure that it accurately reflects the actual characteristics of the USV; (2) introduce multi-source sensor fusion technology to improve the accuracy and robustness of the state estimation; (3) continuously adjust and optimize parameters of algorithms according to the feedback results of field tests.

Future research includes two aspects: on the one hand, this paper proposes the BCS with a dynamically selected binary bit string length strategy, and this scheme can also be used for remote estimation of other autonomous vehicles; on the other hand, we can upgrade the algorithm to estimate all kinds of faults that may exist in USV.

## Figures and Tables

**Table 1:** Estimation performance of case 1.

Estimation Approach	Calculated Load (s)	MSE of the Sway Velocity	MSE of the Yaw Velocity	MSE of the Roll Velocity
Traditional approach	8.950	2.0174	0.8040	58.4744
Our approach	8.574	0.1414	0.2143	3.3266

**Table 2:** Estimation performance of case 2.

Estimation Approach	Calculated Load (s)	MSE of the Sway Velocity	MSE of the Yaw Velocity	MSE of the Roll Velocity
Traditional approach	9.352	0.2558	0.8259	1.6437
Our approach	8.906	0.1414	0.2144	0.6446

## Data Availability

The original contributions presented in the study are included in the article, further inquiries can be directed to the corresponding authors.

## References

[1] Goulon C., Meaux O.L., Vincent-Falquet R., Guillard J. (2021). Hydroacoustic Autonomous boat for Remote fish detection in LakE (HARLE), an unmanned autonomous surface vehicle to monitor fish populations in lakes. Limnol. Oceanogr.-Methods.

[2] Song L., Chen H., Xiong W., Dong Z., Hu K. (2019). Method of Emergency Collision Avoidance for Unmanned Surface Vehicle (USV) based on Motion Ability Database. Pol. Marit. Res..

[3] Xiong Y., Zhu H., Pan L., Wang J. (2022). Research on Intelligent Trajectory Control Method of Water Quality Testing Unmanned Surface Vessel. J. Mar. Sci. Eng..

[4] Abrougui H., Nejim S., Hachicha S., Zaoui C., Dallagi H. (2021). Modeling, parameter identification, guidance and control of an unmanned surface vehicle with experimental results. Ocean Eng..

[5] Cai M., He X., Zhou D. (2022). Performance-improved finite-time fault-tolerant control for linear uncertain systems with intermittent faults: An overshoot suppression strategy. Int. J. Syst. Sci..

[6] Wen P., Li X., Hou N., Mu S. (2022). Distributed recursive fault estimation with binary encoding schemes over sensor networks. Syst. Sci. Control Eng..

[7] Song X., Rong L., Li B., Wang Z., Li J. (2023). Joint state and fault estimation for nonlinear systems with missing measurements and random component faults under Round-Robin Protocol. Int. J. Electr. Power Energy Syst..

[8] Ciuonzo D., Aubry A., Carotenuto V. (2017). Rician MIMO Channel- and Jamming-Aware Decision Fusion. IEEE Trans. Signal Process..

[9] Bao G., Ma L., Yi X. (2022). Recent advances on cooperative control of heterogeneous multi-agent systems subject to constraints: A survey. Syst. Sci. Control Eng..

[10] Li W., Niu Y., Cao Z. (2022). Event-triggered sliding mode control for multi-agent systems subject to channel fading. Int. J. Syst. Sci..

[11] Caballero-Aguila R., Hermoso-Carazo A., Linares-Pérez J. (2022). Networked fusion estimation with multiple uncertainties and time-correlated channel noise. Inf. Fusion.

[12] Wang F., Liang J., Lam J., Yang J., Zhao C. (2023). Robust Filtering for 2-D Systems with Uncertain-Variance Noises and Weighted Try-Once-Discard Protocols. IEEE Trans. Syst. Man Cybern.-Syst..

[13] Li X., Feng S., Hou N., Wang R., Li H., Gao M. (2022). Surface microseismic data denoising based on sparse autoencoder and Kalman filter. Syst. Sci. Control Eng..

[14] Li G., Liu Z., Zhang J., Zheng L. (2019). Robust predictive control for anti-rolling path following of underactuated surface vessels using adaptive Kalman filter. Int. J. Adv. Robot. Syst..

[15] Fei Z., Wang X., Wang Z. (2022). Event-Based Fault Detection for Unmanned Surface Vehicles Subject to Denial-of-Service Attacks. IEEE Trans. Syst. Man Cybern.-Syst..

[16] Wang Y., Han Q. (2016). Network-Based Fault Detection Filter and Controller Coordinated Design for Unmanned Surface Vehicles in Network Environments. IEEE Trans. Ind. Inform..

[17] Sharma S., Sutton R. (2013). A genetic algorithm based nonlinear guidance and control system for an uninhabited surface vehicle. J. Mar. Eng. Technol..

[18] Wang Y., Han Q. (2017). Network-Based Heading Control and Rudder Oscillation Reduction for Unmanned Surface Vehicles. IEEE Trans. Control Syst. Technol..

[19] Zhou X., Zhang Y., Chen J., Zheng X. (2023). A quantized set-membership estimation-based heading control method of unmanned surface vessels under unknown-but-bounded wave-induced disturbances. Asian J. Control.

[20] Chen H., Li Y., Liu C., Xiao Z., Rao H. (2023). Set-Membership State Estimation for Unmanned Surface Vehicle Steering Motion with Try-Once-Discard Protocol. IEEE Sens. J..

[21] Hu J., Yang Y., Liu H., Chen D., Du J. (2020). Non-fragile set-membership estimation for sensor-saturated memristive neural networks via weighted try-once-discard protocol. IET Control Theory Appl..

[22] Yang Y., Hu J., Chen D., Wei Y., Du J. (2020). Non-fragile Suboptimal Set-membership Estimation for Delayed Memristive Neural Networks with Quantization via Maximum-error-first Protocol. Int. J. Control Autom. Syst..

[23] Wu W., Peng Z., Wang D., Liu L., Han Q. (2022). Network-Based Line-of-Sight Path Tracking of Underactuated Unmanned Surface Vehicles with Experiment Results. IEEE Trans. Cybern..

[24] Deng L., Guo T., Wang H., Chi Z., Wu Z., Yuan R. Obstacle Detection of Unmanned Surface Vehicle Based on Lidar Point Cloud Data. Proceedings of the OCEANS 2022.

[25] Gao S., Peng Z., Liu L., Wang D., Han Q. (2023). Fixed-Time Resilient Edge-Triggered Estimation and Control of Surface Vehicles for Cooperative Target Tracking Under Attacks. IEEE Trans. Intell. Veh..

[26] Liu L., Ma L., Zhang F., Bo Y. (2021). Distributed non-fragile set-membership filtering for nonlinear systems under fading channels and bias injection attacks. Int. J. Syst. Sci..

[27] Liu L., Ma L., Wang Y., Zhang J., Bo Y. (2023). Distributed set-membership filtering for time-varying systems under constrained measurements and replay attacks. J. Frankl. Inst.-Eng. Appl. Math..

[28] Li X., Wei G., Wang L. (2021). Distributed set-membership filtering for discrete-time systems subject to denial-of-service attacks and fading measurements: A zonotopic approach. Inf. Sci..

[29] Yi X., Yu H., Wang P., Liu S., Ma L. (2022). Encoding–decoding-based secure filtering for neural networks under mixed attacks. Neurocomputing.

[30] Qu Y., Pang K. (2020). State estimation for a class of artificial neural networks subject to mixed attacks: A set-membership method. Neurocomputing.

[31] Ding D., Wang Z., Han Q., Wei G. (2018). Security Control for Discrete-Time Stochastic Nonlinear Systems Subject to Deception Attacks. IEEE Trans. Syst. Man Cybern.-Syst..

[32] Ding D., Han Q., Xiang Y., Ge H., Zhang X. (2018). A survey on security control and attack detection for industrial cyber-physical systems. Neurocomputing.

[33] Qian W., Wang L., Chen Z. (2018). Local Consensus of Nonlinear Multiagent Systems with Varying Delay Coupling. IEEE Trans. Syst. Man Cybern.-Syst..

[34] Qian W., Gao Y., Yang Y. (2019). Global Consensus of Multiagent Systems with Internal Delays and Communication Delays. IEEE Trans. Syst. Man Cybern.-Syst..

[35] Shen B., Wang Z., Wang D., Luo J., Pu H., Peng Y. (2019). Finite-horizon filtering for a class of nonlinear time-delayed systems with an energy harvesting sensor. Automatica.

[36] Shen B., Wang Z., Tan H. (2018). Guaranteed cost control for uncertain nonlinear systems with mixed time-delays: The discrete-time case. Eur. J. Control.

[37] Dong H., Wang Z., Gao H. (2010). Robust *H_∞_* Filtering for a Class of Nonlinear Networked Systems with Multiple Stochastic Communication Delays and Packet Dropouts. IEEE Trans. Signal Process..

[38] Hu J., Wang Z., Liu S., Gao H. (2016). A variance-constrained approach to recursive state estimation for time-varying complex networks with missing measurements. Automatica.

[39] Hou N., Wang Z., Shen Y., Liu H., Dong H. (2023). A non-fragile approach to fault estimation for a class of multi-rate systems under binary encoding schemes. Int. J. Robust Nonlinear Control.

[40] Gao Y., Ma L., Zhang M., Guo J., Bo Y. (2022). Distributed Set-Membership Filtering for Nonlinear Time-Varying Systems with Dynamic Coding-Decoding Communication Protocol. IEEE Syst. J..

[41] Qu B., Wang Z., Shen B., Dong H., Zhang X. (2024). Secure Particle Filtering with Paillier Encryption–Decryption Scheme: Application to Multi-Machine Power Grids. IEEE Trans. Smart Grid.

[42] Li X., Song J.B., Hou N., Dai D., Yang F. (2023). Finite-horizon distributed set-membership filtering with dynamical bias and DoS attacks under binary encoding schemes. Inf. Sci..

[43] Liu Q., Wang Z. (2021). Moving-Horizon Estimation for Linear Dynamic Networks with Binary Encoding Schemes. IEEE Trans. Autom. Control.

[44] Chen S., Ma L., Ma Y. (2020). Distributed set-membership filtering for nonlinear systems subject to round-robin protocol and stochastic communication protocol over sensor networks. Neurocomputing.

[45] Zou L., Wang Z., Gao H. (2016). Set-membership filtering for time-varying systems with mixed time-delays under Round-Robin and Weighted Try-Once-Discard protocols. Automatica.

[46] Zou L., Wang Z., Shen B., Dong H. (2023). Moving horizon estimation over relay channels: Dealing with packet losses. Automatica.

[47] Hou N., Wang Z., Dong H., Hu J., Liu X. (2022). Sensor Fault Estimation for Nonlinear Complex Networks with Time Delays Under Saturated Innovations: A Binary Encoding Scheme. IEEE Trans. Netw. Sci. Eng..

[48] Liu Q., Wang Z., Dong H., Jiang C. (2022). Remote Estimation for Energy Harvesting Systems Under Multiplicative Noises: A Binary Encoding Scheme with Probabilistic Bit Flips. IEEE Trans. Autom. Control.

[49] Wang S., Wang Z., Dong H., Shen B., Chen Y. (2023). Quadratic filtering for discrete time-varying non-Gaussian systems under binary encoding schemes. Automatica.

[50] Yaz E. (1998). Linear Matrix Inequalities In System And Control Theory. Proc. IEEE.

[51] Ghaoui L., Calafiore G. (2001). Robust filtering for discrete-time systems with bounded noise and parametric uncertainty. IEEE Trans. Autom. Control.

[52] Milanese M., Vicino A. (1991). Estimation theory for nonlinear models and set membership uncertainty. Automatica.

